# A Novel *STAT3* Mutation in a Qatari Patient With Hyper-IgE Syndrome

**DOI:** 10.3389/fped.2019.00130

**Published:** 2019-04-24

**Authors:** Natalia S. Chaimowitz, Justin Branch, Anaid Reyes, Alexander Vargas-Hernández, Jordan S. Orange, Lisa R. Forbes, Mohammed Ehlayel, Saleema C. Purayil, Maryam Ali Al-Nesf, Tiphanie P. Vogel

**Affiliations:** ^1^Section of Immunology, Allergy and Rheumatology, Department of Pediatrics, Center for Human Immunobiology, Baylor College of Medicine, Texas Children's Hospital, Houston, TX, United States; ^2^Allergy and Immunology Section, Hamad Medical Corporation, Doha, Qatar

**Keywords:** STAT3, Job syndrome, AD-HIES, eczema, pneumonia, case report

## Abstract

Autosomal dominant hyper-IgE syndrome caused by mutations in the transcription factor STAT3 (AD-HIES) is characterized by a collection of immunologic and non-immune features including eczema, recurrent infections, elevated IgE levels, and connective tissue anomalies. We report the case of a Qatari child with a history of recurrent staphylococcal skin infections since infancy, who was found to have a novel, *de novo* mutation in STAT3 (c.1934T>A, p.L645Q). The absence of mucocutaneous candidiasis and undetectable IgE levels until the age of 7 years prolonged the time to molecular confirmation of the cause for the patient's immune deficiency. STAT3 p.L645Q was found to have decreased transcriptional capacity. The patient also had low levels of Th17 cells and STAT3 phosphorylation was impaired in patient-derived cells. Nearly 100 unique mutations in STAT3 have been reported in association with AD-HIES.

## Introduction

Autosomal dominant hyper-IgE syndrome (AD-HIES) is a rare primary immunodeficiency conventionally characterized by the triad of eczema, recurrent skin and sinopulmonary infections, and elevated serum IgE. Non-immunological features of AD-HIES can include bone fractures secondary to minor trauma, delayed shedding of deciduous teeth, joint hyperflexibility, and vascular anomalies (1).

While this syndrome was first reported in the 1960s (2) and associated with elevated IgE levels in the 1970s (3), the genetic basis for this disorder was not described until 2007 (4, 5). AD-HIES is caused by heterozygous, germline mutations in the gene for *signal transducer and activator of transcription 3 (STAT3)*. STAT3 is a transcription factor activated downstream of numerous cytokine signals including interleukin (IL)-6, interferon-α and IL-10, among others (6). A consistent immunophenotype in patients with AD-HIES is impaired development of Th17 lymphocytes, mainly due to the key role of cytokine signaling through STAT3 in their generation (7).

Herein, we report a patient with dermatitis and recurrent skin and pulmonary infections found to have a previously unreported, *de novo* mutation in *STAT3* as the cause of AD-HIES.

## Case Report

The patient is a now 15-year-old Arab-Qatari male born full term with no complications, the fifth of six children of a non-consanguineous union. At 8 days of life, he developed diffuse cutaneous pustules starting in the groin and then spreading across the body. The lesions failed to improve with topical antibiotics necessitating hospitalization at age 44 days for intravenous (iv) antibiotics and incision and drainage (I&D). He was diagnosed with infantile eczema and a superimposed bacterial infection; wound cultures were positive for *Staphylococcus aureus*.

**Figure 1:**
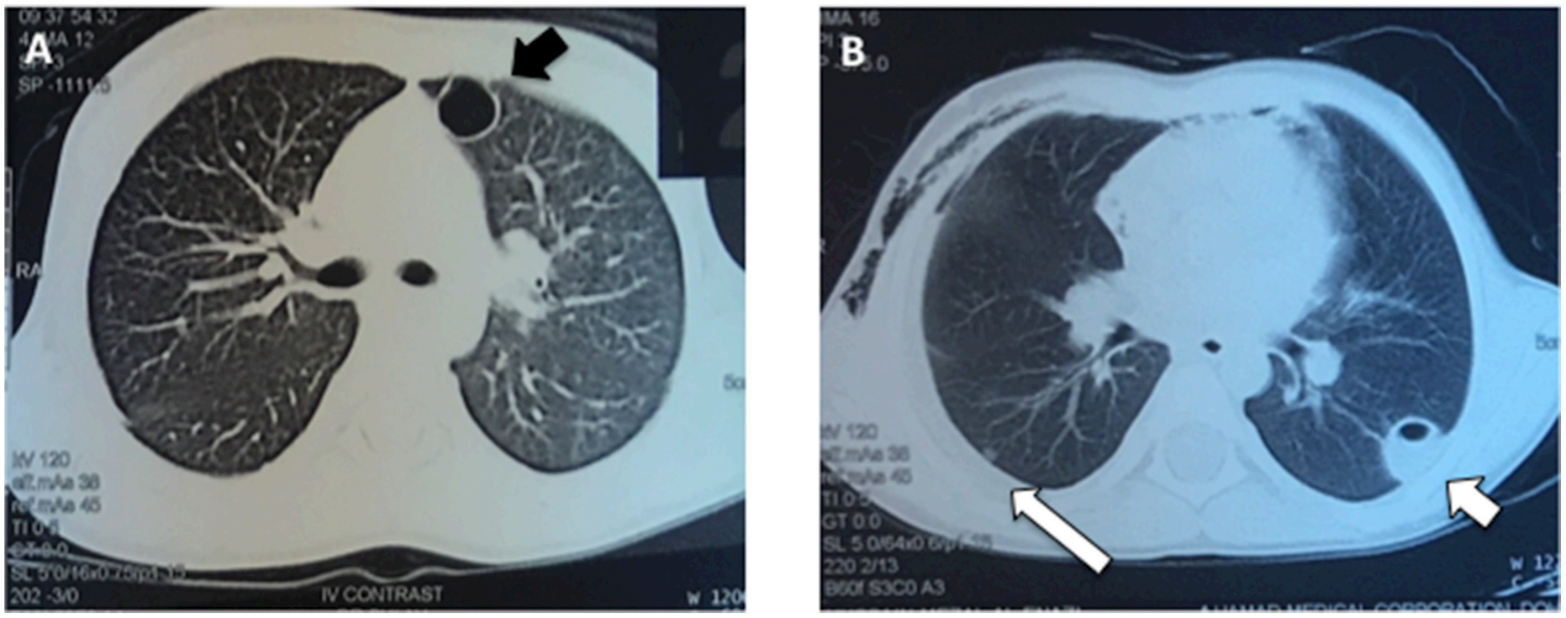
Pulmonary imaging during an acute respiratory infection at age 5 years. **(A)** The patient had numerous, enhancing cavitary lung lesions (black arrow) noted using CT scan of the chest, which improved after drainage and prolonged antibiotic therapy. **(B)** Several of the pneumatoceles were fluid-filled (short white arrow) and a parapneumonic effusion was present (long white arrow).

At 4 months of age, the patient was again admitted to the hospital with a recurrent abscess requiring I&D and iv antibiotics. Throughout his early childhood he continued to develop recurrent skin and soft tissue infections almost monthly and usually without fevers, including at 4 years of age when he had another I&D of a facial abscess with cultures growing methicillin resistant *Staphylococcus aureus*. At age 5 he was admitted to the hospital with pneumonia complicated by a parapneumonic effusion and multiple cavitary lung lesions (Figure 1). He was treated with drainage of the lung nodules and prolonged iv antibiotics.

Given his recurrent infections at an early age of onset requiring repeated hospitalizations and iv treatments, the immunology service was consulted. The patient had several immune evaluations at age 2 and 4 that were normal and included immunoglobulin levels with undetectable IgE and normal lymphocyte subsets, CD11 and CD18 expression, nitroblue tetrazolium testing and myeloperoxidase staining.

**Figure 2:**
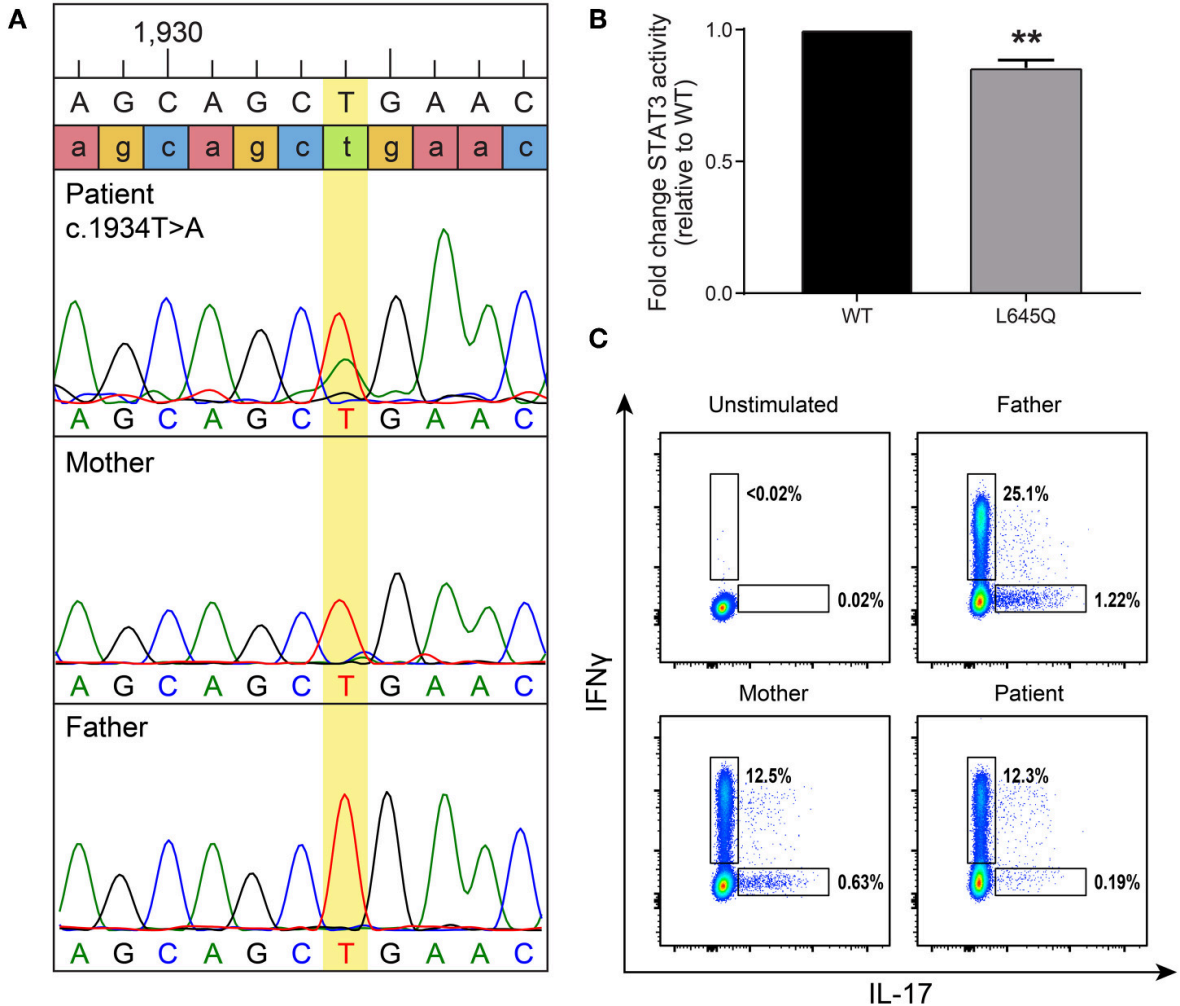
A *de novo* mutation in *STAT3* has decreased basal activity and leads to low Th17 cells. **(A)** Targeted Sanger sequencing revealed a *STAT3* mutation, c.1934T>A (leading to p.L645Q), in the patient that was not present in his parents. **(B)** To measure basal levels of STAT3 activity a luciferase assay was performed. Wild-type (WT, black) or mutant (gray) STAT3 plasmids were co-transfected into STAT3-deficient cells along with a STAT3-responsive luciferase reporter. STAT3 activity is shown as a ratio of firefly/control normalized to WT. Data represent 3 experiments ± SEM, **p < 0.01 as it designates the level of significance (unpaired *t*-test). **(C)** Intracellular cytokine staining was performed to detect Th17 cells produced after stimulation. A gate was placed on CD3+CD4+ T-cells, then they were assessed for the ability to produce IL-17 and interferon-γ (IFNγ) as a positive control. The patient produced reduced levels of Th17 cells compared to his parents.

At 7 years of age, the patient was admitted to the hospital with superinfection of his eczema lesions and his IgE was then found to be elevated at 4,409 kU/L (normal 0–63 kU/L). Given his history of recurrent staphylococcal infections and elevated IgE, AD-HIES was suspected. A *de novo*, heterozygous missense mutation in *STAT3* (c.1934T>A, p.L645Q) was detected (Figure 2A). This novel mutation is located in the SH2 domain, adjacent to amino acids previously reported to contain *STAT3* mutations associated with AD-HIES. It is not reported in publically available databases.

**Figure 3:**
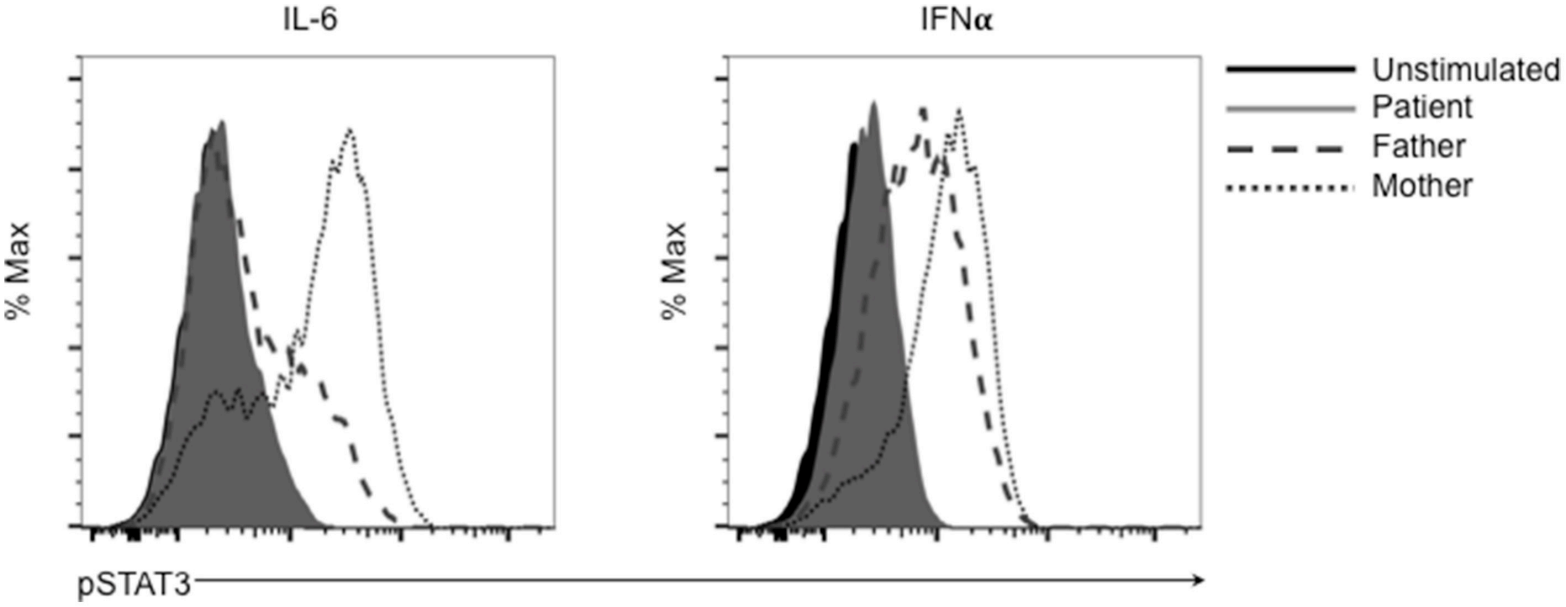
The SH2 domain STAT3 variant p.L645Q fails to phosphorylate normally after stimulation. Patient-derived cells were left unstimulated (solid black) or stimulated with IL-6 or interferon-α (IFNα) and assessed for phospho-STAT3 (p-STAT3) using flow cytometry. Cells derived from the patient (solid gray) fail to phosphorylate after stimulation compared to cells derived from the father (dashed gray) or mother (dotted gray).

This mutation changes a polar glutamine for a non-polar leucine. Reduction of transcriptional activity of STAT3 p.L645Q to 85% of wild-type was confirmed using a luciferase assay (Figure 2B). The patient also had decreased CD3+CD4+ T cells expressing IL-17 (Figure 2C). Furthermore, STAT3 phosphorylation in patient-derived cells was impaired in response to cytokine stimulation (Figure 3), which has been reported in patients with AD-HIES and *STAT3* mutations in the SH2 domain (8).

The patient did not have a history of mucocutaneous candidiasis. He did have eosinophilia (10.7%, 560 cells/μL), a common finding in AD-HIES. His peak serum IgE has been 10,665 kU/L. At 14 years of age, the patient sustained a non-displaced fracture in his ankle following a ground level fall. Bone densitometry showed osteopenia. He did not manifest other non-immunologic features of AD-HIES such as coarse facies, high-arched palate, scoliosis or joint hyper-extensibility, although he was noted to have retained primary upper incisors. He did have two separate and resolving episodes of facial nerve palsy at age 5 and 15 years not clearly associated with a viral infection.

After the diagnosis of AD-HIES, the patient was started on prophylactic cephalexin but continued to struggle with recurrent abscesses. After his response to pneumococcal vaccination was judged insufficient, his prophylactic antibiotic was changed to levofloxacin and he was started on immunoglobulin replacement therapy. He has subsequently experienced a significant decrease in the frequency of his infections and improved quality of life.

## Methods

### Patients

The study was approved by the Baylor College of Medicine Institutional Review Board. All subjects gave written informed consent in accordance with the Declaration of Helsinki. Written informed consent was obtained from the parents of the patient for the publication of this case report and any potentially identifying information/images. Genomic DNA was extracted from whole blood and sent for targeted sequencing of STAT3.

### STAT3 Luciferase

An expression plasmid with the patient's mutation was generated using a plasmid containing the wild-type (WT) STAT3 complementary DNA (cDNA) (NM_139276; Origene) altered with QuikChange site-directed mutagenesis (Agilent Technologies) using the primers: Forward: 5′-GCA AAT GAC ATG TTG TTC TGC TGC TGC TTT GTG TAT G-3′ and Reverse: 5′-CAT ACA CAA AGC AGC AGC AGA ACA ACA TGT CAT TTG C-3′. For STAT3 luciferase studies, STAT3-deficient A4 cells were co-transfected (Lipofetamine 2000, ThermoFisher) with 250 ng of mutant or WT STAT3 plasmid and a firefly luciferase plasmid under STAT3 transcriptional control with a constitutively expressed renilla luciferase (Cignal STAT3, Qiagen). Firefly and renilla levels were measured after 48 h (Dual-Luciferase Reporter Assay, Promega).

### Th17 Detection

Peripheral blood mononuclear cells (PBMCs) from the patient and his parents were isolated using density centrifugation. PBMCs were resuspended in RPMI-1640 media supplemented with 10% FCS, penicillin, streptomycin, and L-glutamine and stimulated overnight using 50 ng/mL PMA and 500 ng/mL calcimycin in the presence of brefeldin A (at 1:1,000 dilution). Flow cytometric analysis of intracellular IL-17 using a BD FACSCanto II was performed after fixation and permeabilization using Cytofix/Cytoperm (BD Biosciences) per the manufacturer's instructions. Antibodies used (BD Biosciences) were anti-CD3-APC-Cy7 (SK7), anti-CD4-PerCP (RPA-T4), anti-interferon-γ-PE (B27) and anti-IL-17-FITC (N49-653). Data were analyzed using FlowJo software.

### Phospho-STAT3 Assay

Immortalized lymphoblastoid cell lines were generated from PBMCs using standard methods. Patient-derived cells (6 x 10^5^) in the log phase of growth were left unstimulated or stimulated for 15 min with IL-6 (10 ng/mL) or interferon-α (50 ng/mL). Cells were then fixed in Cytofix and permeabilized using Perm Buffer III (both BD Biosciences) per the manufacturer's instructions. Cells were incubated with phospho-STAT3 (Y705) antibodies (BD Biosciences) for 1 h at room temperature followed by flow cytometric analysis (BD FACSCanto II). Data were analyzed using FlowJo software.

## Discussion

Mutations in *STAT3* leading to a loss in transcriptional function are the genetic cause of AD-HIES. Here we describe a patient with a novel, *de novo*, heterozygous missense *STAT3* mutation in the SH2 domain, p.L645Q. This mutation was experimentally confirmed to have a reduction in transcriptional capacity (Figure 2B) and led to poor phosphorylation after stimulation (Figure 3).

The clinical phenotype of our patient, recurrent and severe staphylococcal infections starting in infancy, was concerning for a primary immunodeficiency, particularly AD-HIES, despite the initial undetectable IgE level and lack of fungal infections. The pathophysiology of IgE elevation in AD-HIES is poorly understood. An elevated IgE level could be suggestive of a *STAT3* mutation, but a normal IgE level does not exclude the diagnosis, particularly in infants and children (9, 10). However, as demonstrated in this case, an undetectable, or even a normal, IgE level can lead to a diagnostic delay in a patient with a narrow infectious phenotype and few non-immunologic features of AD-HIES.

**Figure 4:**
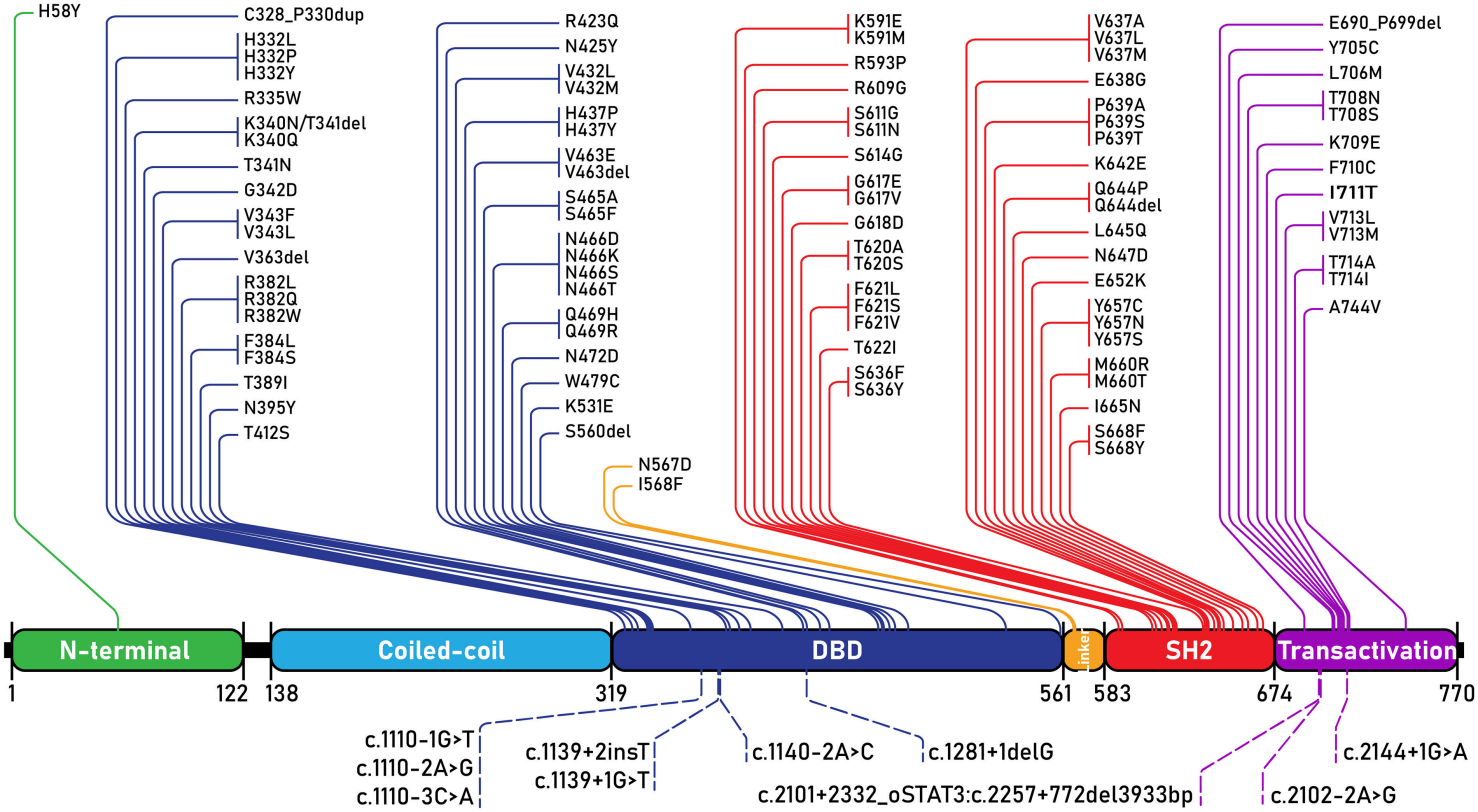
STAT3 mutations associated with AD-HIES. All AD-HIES variants published with a clinical phenotype, represented by domain (1, 11–21). Mutations are separated with coding region mutations above, and non-coding region mutations below. The numbers below the domains represent amino acid locations. DBD, DNA binding domain.

Since *STAT3* mutations were discovered as the genetic basis of AD-HIES, nearly a hundred individual *STAT3* mutations have been reported, with supportive clinical phenotypes, to lead to AD-HIES (Figure 4). AD-HIES associated *STAT3* mutations span the domains of the protein, with the exception of the coiled-coil domain. Gain-of-function (GOF) mutations in *STAT3*, found in malignancy (somatic) and STAT3 GOF syndrome (germline) are found in all domains (1). While few AD-HIES associated *STAT3* mutations are reported outside of the DNA binding and SH2 domains, both these domains are equally represented.

The clinical ability to perform diagnostic genetic testing has dramatically increased. Therefore, it is important for patient evaluation and subsequent treatment to continue to functionally confirm and report novel mutations in known disease genes.

## Ethics Statement

All subjects gave written informed consent in accordance with the Declaration of Helsinki. The study was approved by the Baylor College of Medicine Institutional Review Board.

## Author Contributions

NC, JB, and TV: conception or design of the work. JB, AR, AV-H, JO, LF, ME, SP, MA-N, and TV: data collection. JB, AR, and TV: data analysis and interpretation. NC and TV: drafting the article. NC, JB, AR, AV-H, JO, LF, ME, SP, MA-N, and TV: critical revision of the article.

### Conflict of Interest Statement

The authors declare that the research was conducted in the absence of any commercial or financial relationships that could be construed as a potential conflict of interest.
